# Retraction Note: MicroRNA-205-5p inhibits skin cancer cell proliferation and increase drug sensitivity by targeting TNFAIP8

**DOI:** 10.1038/s41598-022-21189-1

**Published:** 2022-09-27

**Authors:** Xinhong Ge, Suryakant Niture, Minghui Lin, Patrice Cagle, P. Andy Li, Deepak Kumar

**Affiliations:** 1 Department of Dermatology, General Hospital of Ningxia Medical University, Yinchuan, 750004 Ningxia Hui Autonomous Region China; 2 Julius L. Chambers Biomedical Biotechnology Research Institute (BBRI), North Carolina Central University, 1801 Fayetteville St., Durham, NC 27707 USA; 3 Department of Respiratory Diseases, The Forth People’s Hospital of Ningxia Hui Autonomous Region, Yinchuan, 750021 Ningxia Hui Autonomous Region China; 4 Department of Pharmaceutical Sciences, Bio‑Manufacturing Research Institute and Technology Enterprise (BRITE), College of Health and Sciences, North Carolina Central University, Durham, NC 27707 USA

Retraction of: *Scientific Reports* 10.1038/s41598-021-85097-6, published online 11 March 2021

The Editors have retracted this Article.

After publication, concerns were raised regarding potential image overlap in the presented Figures. Specifically, high similarity has been observed between the following images:

- Figure 3C HaCaT EV, TNFAIP8 (rotated 90 degrees) and Figure 3F HaCaT TNFAIP8 siRNA;
- Figure 3C A431 EV and Figure 3F A375 TNFAIP8 siRNA;
- Figure 3F A2058 TNFAIP8 siRNA and Figure 5B A2058 NC mimic;
- Figure 3G A375 and A2058 TNFAIP8 siRNA 24 h.

The Authors provided the raw data to address these concerns; however, these data contained further cases of overlap. The Editors therefore no longer have confidence in the presented data.

All Authors agree with the retraction and its wording.

